# General dental practitioner views on the current and future provision of advanced NHS restorative dentistry services: a cross-sectional survey in England

**DOI:** 10.1038/s41415-022-4035-y

**Published:** 2022-03-04

**Authors:** Christopher O´Connor, Francis Bridges-Smith, Ciara Docherty, Northern Dental Practice Based Research Network, Richard Holliday

**Affiliations:** 4141593955001 School of Dental Sciences, Faculty of Medical Sciences, Newcastle University, Newcastle upon Tyne, UK; Newcastle Dental Hospital, Newcastle Hospitals NHS Foundation Trust, Newcastle upon Tyne, UK; 4141593955002 Newcastle Dental Hospital, Newcastle Hospitals NHS Foundation Trust, Newcastle upon Tyne, UK

## Abstract

**Introduction** Advanced NHS restorative dentistry services are an important aspect of patient care. Managed clinical networks (MCNs) have been proposed as a future model of care.

**Aim** To assess general dental practitioners' (GDPs') satisfaction with the current provision of advanced NHS restorative dentistry services and assess their views on MCNs.

**Methods** A self-administered, online survey was distributed by the Northern Dental Practice Based Research Network and was shared on social media.

**Results** In total, 108 responses were received from GDPs working in England; 55% in the North East. GDPs felt current services for periodontics, endodontics, tooth surface loss and temporomandibular disorders were the most important and were most unsatisfied with periodontics, endodontics and tooth surface loss. The most important barriers to current referral practice were previous referral rejections and the return of costly treatment plans. Opinions were favourable towards the proposed MCNs, with one-third of participants feeling they were already suitable to apply to deliver Level 2 services. There was a notable imbalance between sexes, with fewer female dentists (57%) registering interest in joining an MCN compared to their male counterparts (76%) and female dentists also identifying more barriers to join MCNs, including access to suitable training. Recent graduates were least likely to cite a lack of time as a barrier to engaging with MCNs.

**Conclusions** GDPs are currently unsatisfied with advanced NHS restorative dentistry services but appear keen to engage with MCNs. The survey identified important insights which may help healthcare planners develop services.

**Supplementary Information:**

Zusatzmaterial online: Zu diesem Beitrag sind unter 10.1038/s41415-022-4035-y für autorisierte Leser zusätzliche Dateien abrufbar.

## Key points

- Summarises the issues with current advanced NHS restorative dentistry services.
- Identifies the specific clinic areas that are least supported.
- Explores the future options, including managed clinical networks and workforce considerations.

**Supplementary Information:**

Zusatzmaterial online: Zu diesem Beitrag sind unter 10.1038/s41415-022-4035-y für autorisierte Leser zusätzliche Dateien abrufbar.

## Introduction

The vast majority of NHS dentistry occurs in primary care, with £2.9 billion of NHS funding being spent in 2018-2019.^1^ However, occasionally, patients require specialist-level care and traditionally these services are provided by specialists working in hospital settings. This model has several advantages, such as consolidating care in centres of excellence and training opportunities. However, it also has several potential challenges, such as limited access, inflexibility and perceived barriers between different care settings.

In 2015, NHS England started publishing commissioning standards for the different dental specialties.^2^ These are designed as guides for healthcare commissioners who might be managing funding for several different clinical fields and may not be experts in dentistry. The standards provide guidance around what and how specialist level care should be provided. An important aspect of these is that care complexity is considered in three levels:

- Level 1 care - requires skills that would be expected of all dentists (after completion of vocational training/dental foundation training)
- Level 2 care - requires enhanced skills (but not necessarily on a specialist list)
- Level 3 care - requires management by a specialist or consultant.

The overall aim of the guides and in keeping with the NHS Long Term Plan, is to provide more joined-up care, with greater consistency and accessibility and to break down the barriers between care providers, improving the patient pathway. In order to achieve this, they suggest novel methods of working such as managed clinical networks (MCNs), which aim to unlock structural and cultural barriers.^3^ MCNs are more than a group of clinicians interested in a topic, they are directly involved in managing referrals and providing care. They are managed by NHS England and will include a funded consultant chairperson. The MCN should ensure referrals are triaged and assessed appropriately and aim to link up the patient with the most appropriate local provider. The MCN could include several local, experienced general dental practitioners (GDPs) who have been commissioned to deliver certain Level 2 or Level 3 services within their practices or at designated hubs. The MCN will have robust quality assurance systems in place and GDPs will be able to arrange for consultant/specialist support when required. The basic premise is that the patient is seen by the right person, in the right place and at the right time. In reality, this might involve a specialist or consultant triaging referrals and where appropriate, some aspects of care being allocated to a 'Level 2' provider. For example, for the advanced management of tooth wear involving a full mouth rehabilitation, a GDP with enhanced skills could be commissioned to deliver this service. They would be paid on a sessional or case basis and have access to a specialist for support as required.

Restorative dentistry, given its breath and complexity, provides particular challenges and it is perhaps unsurprising that the 'Commissioning standard for restorative dentistry' was the final speciality to be published in the summer of 2019.^4^ At the current time, there are very few true MCNs in restorative dentistry in England. GDPs will be fundamental to the success, or otherwise, of these new models of working. This study aimed to utilise a Dental Practice Based Research Network in the North of England (https://blogs.ncl.ac.uk/northerndentres/) to gauge the current views of GDPs on this topic. Specifically, we aimed to: assess the satisfaction of GDPs with current provision of advanced NHS restorative dentistry and identify barriers to care; assess awareness of the NHS restorative dentistry commissioning standards; and to explore opinions and willingness to engage with MCNs.

## Materials and methods

### Study cohort

The study design was a self-administered, online, questionnaire survey of GDPs. Ethical approval was obtained from the Newcastle University Research Ethics Committee (Reference number: 308/2020). Consent for participation and use of their anonymous data in this research was obtained. Convenience sampling was used with the Northern Dental Practice Based Research Network (NDPBRN) being the primary distributor (approximately 100 contacts on mailing list). The NDPBRN was formed in 2018 and is based in the North East of England. The survey link was also shared on social media (Twitter and Facebook). The survey was open between 20 January 2020 and 6 April 2020. The last survey response was received on the 18 March 2020 and as such, represents the opinion of the profession before the COVID-19 pandemic.

### Questionnaire

The questionnaire consisted of 17 questions which including sub-questions and free-text areas, totalling 70 research items in total. A copy of the questionnaire is available in the online supplementary information. Initial questions obtained demographic information about the respondents including: sex, ethnicity, work geographical location, units of dental activity (UDA) commitment and date of primary dental degree graduation. Questions next explored normal referral practices for NHS advanced restorative dentistry care and opinions on current care provision and patient pathways. Further questions explored awareness of MCNs, self-rated suitability for Level 2 roles and incentives and barriers to engaging with MCNs. Questions included a mixture of multiple choice and Likert responses, with free-text options available for the questions on satisfaction with current referral services, barriers to current referral practices, justification of Level 2 eligibility and incentives to MCN engagement. The survey was conducted using online survey software (Online Surveys, Jisc, Bristol, UK). The survey was piloted with eight members of the NDPBRN management committee and was refined following feedback.

### Data analysis

Statistical Package for the Social Sciences (IBM Corp, released 2019. IBM SPSS Statistics for Windows, version 26.0. Armonk, NY: IBM Corp) was used to analyse data. Descriptive statistics were calculated for each variable. To allow easy comparison the response data were grouped into dichotomous variables, for example respondents who agreed with a statement ('strongly agreed' or 'agreed') and those that did not (all other responses). The conditions for the dichotomous variables are described within in the results section when implemented.

Chi-square tests (Fischer's exact test when observed items <5) were used to explore the grouped data further. The independent variables we explored were: sex, year of graduation (before 2010 vs after 2010) and area of work (North East vs other areas in England). Cross tabs and Chi-square tests were also completed between the independent variables to help check for confounding factors in the data set.

## Results

**Fig. 1:**
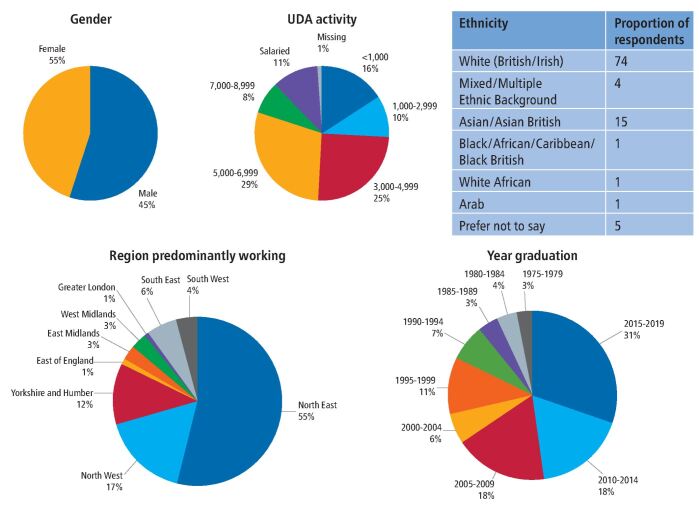
Demographic data collected from questionnaire

A total of 118 respondents completed the survey. Of the respondents, ten were removed as they were not working predominantly in England. The demographics of the respondents are presented in Figure 1. Dentists' views were represented from across the whole of England but the North East region was disproportionately represented, making up 55% of the respondents. The survey was most likely to be completed by more recent graduates, with graduates between 2010-2019 making up 48% of all respondents. None of the dentists who completed the survey had a UDA activity >8,999 per annum but there was considerable variation in the number completed and 11% of respondents were in salaried positions. From an ethnicity standpoint, the respondents appeared to be representative of the national make up of General Dental Council (GDC) registrants.^5^

Dentist were asked to indicate how often they referred for advanced NHS services across a range of subgroups that make up restorative dentistry. They were also asked to rate the importance of advanced NHS support for these subgroups and finally were asked to indicate how satisfied they were with the current advanced NHS provisions for these referrals. The results of these three questions are combined in Table 1. The results show that dentists are most likely to send at least one temporomandibular disorder (TMD) referral to advanced NHS services in a three-month period (55% of all respondents) and are least likely to send a fixed prosthodontic referral (7% of all respondents). In total, 93% of all respondents rated advanced NHS provision for periodontal disease as 'very important' or 'important' which was the highest percentage of all the referral types, closely followed by endodontics (90%), tooth surface loss (87%) and TMD (86%). Advanced NHS provision for implants was rated as important by the fewest dentist in the survey (46%) followed by fixed prosthodontics (60%) and removable prosthodontics (68%).

**Table 1:** Responses to questions relating to current referral practice to advanced NHS services for restorative dentistry sub-specialities and opinions on the importance of and satisfaction with these services

Referral type	Q9) Over three months, on average, how many referrals do you submit for advanced restorative dentistry NHS provision?		Q10) How important do you think the advanced NHS service provision is for the following conditions?		Q11) How satisfied are you with the current advanced NHS support for the following conditions?		
	At least one referral^*^ (%)	Missing (%)	Rated important^**^ (%)	Missing (%)	Unsatisfied^†^ (%)	N/A rarely refer (%)	Missing (%)
TMD	55	2	86	0	33	10	1
Anxiety/psychosocial issues	50	2	81	0	39	17	0
Periodontal disease	49	3	93	1	66	8	0
Endodontics	49	3	90	1	65	8	0
Tooth surface loss	44	5	87	1	50	15	0
Complex medical history	44	2	85	0	30	16	1
Removable prosthodontics	17	4	68	1	41	25	0
Implants	11	6	46	3	38	26	0
Fixed prosthodontics	7	5	60	2	38	31	0
Key: * = Respondents ticked either (1-3), (4-6), (7-9) or (10+) in response to the question: 'over three months, on average, how many referrals do you submit for advanced restorative dentistry NHS provision?' ** = Respondents either rated the referral type 'very important' or 'important' on a five-point scale † = Respondents either scored 'very unsatisfied' or 'unsatisfied' on a six-point scale ('n/a rarely refer' being a sixth option)							

Dentists responding to this survey were most unsatisfied with the advanced NHS support received for periodontal diseases, with 66% of all respondents indicating they were 'unsatisfied' or 'very unsatisfied' with the current service. In the survey, respondents were also given the option to select if they couldn't judge the current advanced NHS support received, as they referred so rarely (in the case of periodontal disease this was 8% of respondents). If these respondents are removed from the analysis, then of the 99 dentists that rated their satisfaction of advanced NHS provision of periodontal care, 72% (n = 71) rated as 'very unsatisfactory' or 'unsatisfactory.'

The reason for this dissatisfaction is explored in Table 2 where dentists were asked to rate (on a five-point Likert-like scale) how significant certain barriers were to their current referral practices. The survey also received 64 free-text comments related to both the levels of satisfaction regarding advanced NHS provision and the barriers to referring. A selection of these free-text comments has been included in Table 2 to illustrate the views expressed in the survey which were, without exception, critical of the current advanced NHS provision.

**Table 2:** Responses to questions about potential barriers to current referral practice

How significant are the following barriers to your current referral practice?			
Barrier	Significant barrier^*^ (%)	Missing (%)	Selected free-text comments
Referral rejection	89	1	'No confidence referral system can cope with appropriate referrals, therefore it feels like patients are rejected for the wrong motives'
			'Referrals are bounced back for no real reason and there is no consistency'
			'Rarely refer because when I do it doesn't get accepted and I get bad mouthed by the [redacted] staff about why it's not acceptable'
			'The culture at [redacted] appears to be "why should we not accept this referral" as their main purpose. It means having a referral accepted is almost impossible'
Costly treatment plans returned	71	1	'Patients are often returned following consultations with outrageous treatment plans which would either have financial loss to practices and associate dentists or are unsuitable and require the treatment to be completed by a specialist'
			'We have had treatment plans back which would cost £5,000 and would take 20 hours to complete. We have 15,000 other patients to treat also and cannot spend this amount of time or money (£1,500 lab bills) on one patient!'
Previous experience	51	1	'I rarely refer if I can, quite simply because the service is so unhelpful'
			'Given that I rarely refer to a dental hospital in the first place (given the travel required from the area where I work), the % of rejected referrals or overly complex treatment plans received makes the whole process a waste of time'
Unclear referral pathways	50	1	'For my patients living in [redacted] there is an endo referral pathway but not [redacted]. This is frustrating!'
			'No criteria given for what referrals require before [redacted] accepts case'
Time-consuming referral	40	2	'The forms are also far too long and complicated'
			'We now have to fill in a PDF form and that does take time even when using patient demographics. Attaching radiographs can be a pain...I am regularly getting home late because of referrals'
Key: * = Respondents either rated the referral type 'very significant' or 'significant' on a five-point scale			

In total, 50% (n = 54) of all respondents had not heard of the new commissioning standard for restorative dentistry but (after brief explanation within the survey) 89% of respondents strongly agreed (n = 51) or agreed (n = 45) that MCNs would be beneficial for NHS patients and 93% agreed that they would be beneficial to GDPs. Overall, 68% of respondents 'strongly agreed' (n = 41) or 'agreed' (n = 32) that they would be interested in participating in a restorative dentistry MCN and 33% of respondents felt that they were already suitable to apply for a Level 2 role within such a network.

The responses to potential incentives and barriers to MCNs are explored in Table 3. In total, 94% of respondents felt that 'access to specialist colleagues' was an important incentive for being part of a MCN. This was the incentive rated important by most respondents, followed closely by 'monetary incentive (92%)' and 'professional development' (89%). The barriers that most dentists agreed were significant barriers to joining a MCN were 'access to appropriate training (65%) and 'insufficient skill' (60%).

**Table 3:** Responses to questions about potential incentives and barriers to considering a role in a MCN

How important would the following incentives and barriers be for you when considering a role in a MCN?					
Incentives	Important^*^ (%)	Missing (%)	Barrier	Significant barrier^**^ (%)	Missing (%)
Access to specialist colleagues	94	0	Access to appropriate training	65	2
Monetary incentive	92	0	Insufficient skill	60	0
Professional development	89	0	Already too busy	52	1
Further education	86	0	Too much additional responsibility	38	1
Guaranteed patient flow	78	0	Not interested	18	1
Key: * = Respondents either rated the referral type 'very important' or 'important' on a five-point scale ** = Respondents either scored 'very significant barrier' or 'significant barrier' on a five-point scale					

One of the themes that emerged in the free-text comments was the issue of responsibility. Respondents felt that the 'overall responsibility [for care was] with the consultant' and that Level 2 dentists would have access to the 'same level of indemnity protection that hospital providers get'.

Differences in responses between sex, year of graduation and location of work were explored for all grouped responses. All questions that showed significantly different responses are listed along with their cross tabulations in the online supplementary information (an example is given in Table 4). One of the key differences found was that 57% (28 out of 49) of female dentists were interested in participating in a MCN compared to 76% (45 out of 59) of male dentists, which was a significant difference (P = 0.034). They were also significantly (P = 0.003) less likely to feel already suitable for a Level 2 roles, with 18% of women responding positively compared to 46% of men. Finally, women were more likely to report feeling that 'insufficient skill' (P = 0.030) and 'access to appropriate training' (P = 0.029) were significant barriers to taking part in a MCN.

**Table 4:** Cross tabulation comparing the responses between sexes to Question 14.3

Question 14.3		I would be interested in participating in a MCN		Total
		Agree^*^	Don't agree^**^	
Sex	Male	45	14	59
	Female	28	21	49
**Total**		**73**	**35**	**108**
Key: * = Agree is a composite score of responses 'strongly agree' and 'agree' ** = Don't agree is a composite score of responses 'neither agree nor disagree', 'disagree' and 'strongly disagree' Pearson chi-square value: P = 0.034				

More recent graduates (after 2010) were significantly more likely to feel satisfied with the current advanced NHS services for implants, endodontics, tooth surface loss and removable prosthodontics. Only 19% of recent graduates felt they were already suitable for a Level 2 role within a restorative dentistry MCN, which was significantly fewer (0.035) compared to 46% of older graduates. Recent graduates were significantly (P = 0.027) less likely to feel that 'being already too busy' was a significant barrier to joining a restorative dentistry MCN but were more likely to feel that 'insufficient skill' (P <0.001) and 'access to appropriate training' (P = 0.020) were.

Dentists in the North East were significantly more satisfied with advanced NHS services in implants, endodontics, removable prosthodontics, TMD and anxiety and psychosocial issues. They were also significantly more likely to agree that MCNs would be beneficial for NHS patients (P = 0.029), GDPs (P = 0.004) and were more interested in taking part in a restorative dentistry MCN (P = 0.011).

Regarding potential confounding factors, these were all found to be non-significant: 55% of female respondents were recently graduated compared to 42% of male respondents (P = 0.187); 53% of female respondents worked predominantly in the North East compared to 56% of their male counterparts (P = 0.77); and 60% of recent graduates worked in the North East compared to 50% of older graduates (P = 0.31).

## Discussion

The results of this survey suggest that there is a strong feeling among the surveyed GDPs that the current provision of advanced NHS restorative dentistry services is unsatisfactory. The changes outlined within the NHS England 'Commissioning standards for restorative dentistry'^4^ appear to be welcomed by the vast majority of GDPs and there are a number of NHS providers who consider themselves already able to apply and complete a Level 2 role. There were notable imbalances between men and women, with women reporting that they would be less likely to engage with MCNs and rarely feeling they had the enhanced skills, or access to training, to provide Level 2 services. Access to specialist colleagues, appropriate remuneration and training were identified as important to the success of a MCN.

### How this related to previous research

Previous research on the views of GDP referrers to existing advanced NHS restorative dentistry services have found similar themes. For example, a study focusing on periodontics in the North East of England in 2007 identified similar issues, with some GDPs feeling that the dental hospital would not do anything differently.^6^ The research identified that features of an ideal service were accessibility, reputation and communication. A report, including interviews with GDPs in Scotland, explored perceptions towards restorative dentistry services.^7^ In keeping with our findings, the interviews identified that GDPs felt referral pathways were unclear and sometimes they were asked to deliver treatment plans that they were unable to provide.

To the best of our knowledge, this is the first published work investigating GDPs' awareness and opinions of MCNs for restorative dentistry, which is expected given the relatively short time since the restorative dentistry standards were published. Previous literature from other specialities has focused on the delivery of services and the quality of referrals from the perspective of the commissioner or consultant specialist and the views of the referring GDPs has rarely been captured in any detail.^8^^,^^9^^,^^10^

Differences between men and women were an important factor identified by our survey, with women less likely to self-identify as already at Level 2 and more likely to identify insufficient skill and access to training as barriers to participating in a restorative dentistry MCN. As of December 2020, 27% of registrants on the GDC speciality lists for restorative dentistry, endodontics, periodontics and prosthodontics were women.^11^ This shows a similar pattern to the data from our survey with women being underrepresented in these 'specialist' roles.

### Limitations

The results of this study should be interpreted with caution because of the relatively small sample size. Dentists working in or near the North East of England are disproportionately represented in the survey results and the profile of these respondents may be different to those from other areas of England; they are more likely to have received the questionnaire directly from the NDPBRN rather than via social media channels. Also, while the results of the survey show significant differences between different groups of dentists, this could have been caused by a number of confounding factors. Like all open online surveys, there is an inherit risk of selection bias in respondents and also survey fraud. It is also important to identify that questionnaire-based studies have several general limitations such as oversimplification of issues/concepts, leading respondents and selection bias. Qualitative research techniques such as individual interviews or focus groups could have provided much richer data and should be considered in the future.

It is also important to acknowledge that this survey was conducted immediately before the COVID-19 pandemic and thus represents the views of the dental community before this event, which has changed the landscape of dentistry in England, including the provision of advanced NHS restorative dentistry services. Restorative dentistry is a broad speciality and this survey only focused on the referral role (from primary care) and did not directly consider the multi-disciplinary services provided, such as the management of head and neck oncology or developmental conditions (cleft lip and palate, hypodontia, amelogenesis imperfecta, dentinogenesis imperfecta).

### Implications for future work

There is urgent need to explore some of the themes that have emerged within this survey so that healthcare planners can elicit if the changes implemented in the commissioning standards address and improve satisfaction of GDPs with advanced NHS provision. This is also an ideal time to compare and contrast the effectiveness of existing and newly-formed MCNs so that the key requirements needed to make them successful can be established.

Unsurprisingly, more recent graduates felt less likely to be able to deliver Level 2 roles but were less likely (compared to older graduates) to cite insufficient time as a barrier to MCN engagement. This potentially identifies an important group of practitioners who could be targeted to engage with MCNs through targeted training opportunities.

This research study was conceived and conducted by a practice-based research network. These networks are ideally placed to deliver research on important real-life questions with a large potential to impact on patient care. The NDPBRN has supported several research projects since its formation, but this is the first project that the network has initiated and conducted itself. The rich data gathered show the value of this approach.

## Conclusions

GDPs were generally unsatisfied with the current provision of advanced NHS restorative dentistry services, being most unsatisfied with referrals for periodontal disease, endodontics and tooth surface loss. Important barriers to current referral practice were previous referral rejections and the return of costly treatment plans. Awareness of the NHS restorative dentistry commissioning standards was low but practitioners were positive to the recommendations and keen to engage with programmes such as MCNs. Many practitioners felt they were able to offer Level 2 services; however, there was a notable imbalance between male and female dentists.

## Supplementary Information

Supplementary Information (PDF 191KB)
