# Supplementary material for: General dental practitioner views on the current and future provision of advanced NHS restorative dentistry services: a cross-sectional survey in England
Source: Br Dent J. 2022 Mar 4:1–6. Online ahead of print. doi: 10.1038/s41415-022-4035-y (PMC8896073; doi:10.1038/s41415-022-4035-y)
Supplement: Supplementary file 1 — Supplementary Information (PDF 191KB) [file 41415_2022_4035_MOESM1_ESM.pdf]

Restorative Dentistry Referrals - GDP views

Page 1: Introduction

Introduction

This survey should take no longer than 20 minutes to complete.

It is an opportunity for General Dental Practitioners (GDPs) to give their views on the current and future provision of advanced Restorative Dentistry services.

It aims to:

- Assess the satisfaction of GDPs with current NHS provision of Restorative Dentistry
- Assess current barriers to NHS referrals for Restorative Dentistry
- Assess GDPs' awareness of NHS restorative commissioning guidelines
- Assess GDPs' opinions on managed clinical networks

Page 2: Consent to participation and data usage

Consent to Participation

I confirm that:

1. I understand that no personal information that could identify me will be collected and that all my responses will be **anonymous**.

2. I voluntarily consent to participation in this research project and understand that once the survey is complete the responses cannot be withdrawn or traced back to the individual.

3. I understand that my survey answers may be quoted directly but without acknowledgement due to the nature of the anonymity of the survey.

4. I understand that the data collected will be deleted on completion of the research project.

5. I confirm that I am a general dental practitioner in the UK registered with the General Dental Council.

Confirmation: ✱ *Required*

- (•) I confirm that I am happy to proceed.

Newcastle University Ethics Committee have reviewed and approved this project (Ref: 308/2020)

Page 3: Demographics

Gender

- Male
- Female
- Other
- Prefer not to say

Age

- 21 - 30
- 31 - 40
- 41 - 50
- 51 - 60
- 61 - 70
- 71+
- Prefer not to say

Ethnicity

- White (British/Irish)
- Mixed/Multiple Ethnic Background
- Asian/Asian British
- Black/African/Caribbean/Black British
- Arab
- Prefer not to say
- Other

If you selected Other, please specify:

Which of the following areas do you work in? (If you work in more than one area, please select the area you carry out *most* of your work in)

- North East
- North West
- Yorkshire and Humber
- East of England
- East Midlands
- West Midlands
- Greater London
- South East
- South West
- Scotland
- Wales
- Northern Ireland

Approximately how many UDAs do you complete per financial year?

- <1000
- 1000 - 2999
- 3000 - 4999
- 5000 - 6999
- 7000 - 8999
- 9000+
- Foundation dentist/salaried

Where did you complete your undergraduate training?

- UK
- Overseas - EU
- Overseas - other

Which dental school did you attend?

- Aberdeen
- Birmingham
- Bristol
- Cardiff
- Dundee
- Glasgow
- King's College London
- Leeds
- Liverpool
- Manchester
- Newcastle
- Plymouth
- Queen Mary, University of London
- Queen's, Belfast
- Sheffield
- UCLan

If you selected Other, please specify:

Please specify

In which year did you graduate from your primary dentistry degree?

- 2015 - 2019
- 2010 - 2014
- 2005 - 2009
- 2000 - 2004
- 1995 - 1999
- 1990 - 1994
- 1985 - 1989
- 1980 - 1984
- 1975 - 1979
- 1970 - 1974
- 1965 - 1969
- Pre - 1965

Page 4: Current Referral Pathways (Restorative Dentistry)

Over 3 months, on average, how many referrals do you submit for advanced restorative NHS provision? e.g. referrals to a dental hospital

Please don't select more than 1 answer(s) per row.

	0	1 - 3	4 - 6	7 - 9	10+
Implants	☐	☐	☐	☐	☐
Endodontics	☐	☐	☐	☐	☐
Periodontal disease	☐	☐	☐	☐	☐
Tooth surface loss	☐	☐	☐	☐	☐
Removable prosthodontics	☐	☐	☐	☐	☐
Fixed prosthodontics	☐	☐	☐	☐	☐
Temporomandibular joint disorder	☐	☐	☐	☐	☐
Complex medical history	☐	☐	☐	☐	☐
Anxiety/psychosocial issues	☐	☐	☐	☐	☐

How **important** do you think advanced NHS service provision is for the following conditions?

Please don't select more than 1 answer(s) per row.

	Very important	Important	Neither important nor unimportant	Unimportant	Very unimportant
Implants	☐	☐	☐	☐	☐
Endodontics	☐	☐	☐	☐	☐
Periodontal disease	☐	☐	☐	☐	☐

Tooth surface loss	☐	☐	☐	☐	☐
Removable prosthodontics	☐	☐	☐	☐	☐
Fixed prosthodontics	☐	☐	☐	☐	☐
Temporomandibular joint disorder	☐	☐	☐	☐	☐
Complex medical history	☐	☐	☐	☐	☐
Anxiety/psychosocial issues	☐	☐	☐	☐	☐

How **satisfied** are you with the **current** advanced NHS support for the following referral categories?

Please don't select more than 1 answer(s) per row.

	Very satisfied	Satisfied	Neither satisfied nor unsatisfied	Unsatisfied	Very unsatisfied	N/a - rarely refer for this treatment
Implants	☐	☐	☐	☐	☐	☐
Endodontics	☐	☐	☐	☐	☐	☐
Periodontal disease	☐	☐	☐	☐	☐	☐
Tooth surface loss	☐	☐	☐	☐	☐	☐
Removable prosthodontics	☐	☐	☐	☐	☐	☐
Fixed prosthodontics	☐	☐	☐	☐	☐	☐
Temporomandibular joint disorder	☐	☐	☐	☐	☐	☐
Complex medical history	☐	☐	☐	☐	☐	☐
Anxiety/psychosocial issues	☐	☐	☐	☐	☐	☐

Please give further information (optional)

Page 5: Barriers to Referral

How significant are the following potential barriers to your current referral practise?

Please don't select more than 1 answer(s) per row.

	No barrier	Very insignificant barrier	Insignificant barrier	Significant barrier	Very significant barrier
Unclear pathway of referral	☐	☐	☐	☐	☐
Referral rejection	☐	☐	☐	☐	☐
Costly treatment plans returned	☐	☐	☐	☐	☐
Previous experience	☐	☐	☐	☐	☐
Time consuming referral	☐	☐	☐	☐	☐

Please provide further details (optional)

Page 6: NHS England Commissioning Standard for Restorative Dentistry

In July 2019, a new 'standard' was published for how future NHS Restorative Dentistry services should be provided.

This document was published by NHS England and called the *'Commissioning Standard for Restorative Dentistry'* .

Please select from the following options:

- I have never heard of this document
- I have briefly discussed this document but have not read it
- I have read this document

Care is divided into 3 levels of complexity:

Level 1 - the minimum standard for performers on the NHS performer list

Level 2 - procedural and/or patient complexity requiring a clinician with enhanced skills and experience who may or may not be on a specialist register

Level 3 - complexity requiring specialist or consultant management

Managed Clinical Networks (MCNs) are proposed as a model for delivering more advanced care. MCNs are networks of skilled GDPs providing level 2 care under the supervision of a Restorative Dentistry consultant. GDPs would be commissioned to provide this service although there maybe different models of remuneration in different regions.

Please consider the following statement:

Please don't select more than 1 answer(s) per row.

	Strongly agree	Agree	Neither agree nor disagree	Disagree	Strongly disagree
MCNs would be beneficial for NHS patients	☐	☐	☐	☐	☐
MCNs would be beneficial to GDPs	☐	☐	☐	☐	☐
I would be interested in participating in a Restorative Dentistry MCN	☐	☐	☐	☐	☐

Do you feel that you are already suitable to apply for a level 2 role?

- ○ Yes
- ○ No
- ○ Maybe

Could you justify this based on your portfolio of clinical experience or formal qualifications? (Please provide brief details of qualifications or ‘clinical experience portfolio’)

How **important** would the following incentives be for you when considering a role in an MCN?

Please don't select more than 1 answer(s) per row.

	Very important	Important	Neither unimportant nor important	Unimportant	Very unimportant
Guaranteed patient flow	☐	☐	☐	☐	☐
Monetary incentive	☐	☐	☐	☐	☐
Further education	☐	☐	☐	☐	☐
Professional development	☐	☐	☐	☐	☐
Access to specialist colleagues	☐	☐	☐	☐	☐

Other:

Are there any other incentives you would require?

How much of a **barrier** would the following issues be for you to take part with an MCN?

Please don't select more than 1 answer(s) per row.

	No barrier	Very insignificant barrier	Insignificant barrier	Significant barrier	Very significant barrier
Insufficient skill	☐	☐	☐	☐	☐

Access to appropriate training	☐	☐	☐	☐	☐
Too much additional responsibility	☐	☐	☐	☐	☐
Already too busy	☐	☐	☐	☐	☐
Not interested	☐	☐	☐	☐	☐

If you would like to read about this further, the full document can be accessed here: https://www.england.nhs.uk/wp-content/uploads/2019/07/commissioning-standard-for-restorative-dentistry-v1.pdf

Page 7: Thank you for your participation

Do you want to take part in future research with the Northern Dental Practice Based Research Network?

Already a network member - keep an eye out for emails?

Not yet a member - sign up today (it's free!)

Exploratory statstics: Gender

Below are all the instances where the independent gender revealed a significant variation in responses for the grouped responses to the questionnaire

**Question 11.3**		**How satisfied are you with the current advanced NHS treatment for periodontal disease**		**Total**
		**Satisfied**	**Not satisfied**	
**Gender**	**Male**	11	44	55
	**Female**	17	27	44
**Total**		28	71	99
Respondents removed from analysis as selected the option n/a - rarely refer for this treatment				8

* satisfied is a composite score of responses 'very satisfied' and 'satisfied'
** not satisfied is a composite score of responses 'neither satisfied nor unsatisfied', 'unsatisfied' and 'very unsatisfied'

Pearson Chi Square: P= 0.041
Implication: Female dentists are significantly more likely to report being satisfied with the advanced NHS treatment of periodontal disease.

**Question 11.7**		How satisfied are you with the current advanced NHS treatment for TMD		Total
		Satisfied	Not satisfied	
Gender	Male	29	25	54
	Female	31	11	42
Total		60	36	96
Respondents removed from analysis as selected the option n/a - rarely refer for this treatment				10

* satisfied is a composite score of responses 'very satisfied' and 'satisfied'
** not satisfied is a composite score of responses 'neither satisfied nor unsatisfied', 'unsatisfied' and 'very unsatisfied'

Pearson Chi Square: P= 0.044
Implication: Female dentists are significantly more likely to report being satisfied with the advanced NHS treatment of TMD.

Question 11.8		How satisfied are you with the current advanced NHS treatment for restorative patients with complex medical histories		Total
		Satisfied	Not satisfied	
Gender	Male	29	23	52
	Female	29	9	38
Total		58	32	90
Respondents removed from analysis as selected the option n/a - rarely refer for this treatment				16

* satisfied is a composite score of responses 'very satisfied' and 'satisfied'
** not satisfied is a composite score of responses 'neither satisfied nor unsatisfied', 'unsatisfied' and 'very unsatisfied'

Pearson Chi Square: P= 0.044
Implication: Female dentists are significantly more likely to be satisfied with the advanced NHS treatment of restorative patients with complex medical histories.

Question 12.5.		How significant a barrier is 'time consuming referral' to your current referral practises		Total
		Significant barrier*	Not a significant barrrier**	
Gender	Male	29	29	58
	Female	14	34	48
Total		43	63	106

* significant barrier is a composite score of responses 'very significant barrier' and 'significant barrier'
** Not a significant barrier is a composite score of responses 'no barrier', 'very insignificant barrier' and 'insignificant barrier'

Pearson Chi Square: P= 0.030
Implication: Male dentists are significantly more likely to think time consuming referral process is a significant barrier to current referral practises

**Question 14.3**		**I would be interested in participating in a MCN**		**Total**
		**Agree**	**Don't agree***	
**Gender**	**Male**	45	14	59
	**Female**	28	21	49
**Total**		73	35	108

*'Agree' is a composite score of responses 'strongly agree' and 'agree'
**'Don't agree' is a composite score of responses 'neither agree nor disagree', 'disagree', 'strongly disagree'.'

Pearson Chi Square value: P= 0.034
Implication: Female dentists are significantly less likely agree that they would like to participate in a MCN

**Question 15**		Do you feel that you are already suitable to apply for a level 2 role?		Total
		Yes	No or Maybe	
Gender	Male	27	32	59
	Female	9	40	49
Total		36	72	108

Pearson Chi Square value: P= 0.003
Implication: Female dentists are significantly less likely to feel already suitable for a level 2 role in a MCN.

**Question 17.1**		How much of a barrier would 'insufficient skill' be for you to take part in a MCN?		Total
		Significant barrier*	Not a significant barrrier**	
Gender	Male	30	29	59
	Female	35	14	49
Total		65	43	108

significant barrier is a composite score of responses 'very significant barrier' and 'significant barrier'
** Not a significant barrier is a composite score of responses 'no barrier', 'very insignificant barrier' and 'insignificant barrier'

Pearson Chi Square value: P= 0.030
Implication: Female dentists are significantly more likely to think 'insufficient skill' is a barrier to them taking part in a MCN

**Question 17.2**		How much of a barrier would ‘access to appropriate training’ be for you to take part in a MCN?		Total
		Significant barrier*	Not a significant barrrier**	
Gender	Male	33	25	58
	Female	37	11	48
Total		70	36	106

significant barrier is a composite score of responses ‘very significant barrier’ and ‘significant barrier’
** Not a significant barrier is a composite score of responses ‘no barrier’, ‘very insignificant barrier’ and ‘insignificant barrier’

Pearson Chi Square value: P= 0.029
Implication: Female dentists are significantly more likely to think ‘insufficient skill’ is a barrier to them taking part in a MCN

Exploratory statistics: Area of work

Below are all the instances where the independent variable ‘area of work’ revealed a significant variationfor the grouped responses to the questionnaire. For the purposes of this analysis area of work work split inmate two groups those that worked predominantly in the North East” and those that worked predominantly in other regions of England.

**Question 9.8**		Over 3 months, on average, how many referrals do you submit for advanced restorative NHS provision for complex medical histories		Total
		0	More than 1*	
Region of work	North East	40	18	58
	Rest of England	18	30	48
Total		58	48	106

** More than 1 is a composite score of responses ‘1-3’, ‘4-6’, ‘7-9’ and ‘10+’.

Pearson Chi Square value: P= 0.001
Implication: Dentists working in the North East are significantly more likely to report referring at least one patient with a complex medical history in a 3 month period to advanced restorative NHS provision.

**Question 11.1**		How satisfied are you with the current advanced NHS treatment for Implants		Total
		Satisfied	Not satisfied	
Region of work	North East	30	17	47
	Rest of England	9	24	33
Total		39	41	80
Respondents removed from analysis as selected the option ’n/a - rarely refer for this treatment’				26

* satisfied is a composite score of responses ‘very satisfied’ and ‘satisfied’
** not satisfied is a composite score of responses ‘neither satisfied nor unsatisfied’, ‘unsatisfied’ and ‘very unsatisfied’

Pearson Chi Square: P= 0.001
Implication: Dentists working in the North East are significantly more likely to report being satisfied with the advanced NHS treatment of implants.

Question 11.2		How satisfied are you with the current advanced NHS treatment for endodontics		Total
		Satisfied	Not satisfied	
Region of work	North East	24	30	54
	Rest of England	5	40	45
Total		29	70	99
Respondents removed from analysis as selected the option 'n/a - rarely refer for this treatment'				8

* satisfied is a composite score of responses 'very satisfied' and 'satisfied'
** not satisfied is a composite score of responses 'neither satisfied nor unsatisfied', 'unsatisfied' and 'very unsatisfied'

Pearson Chi Square: P= < 0.001
Implication: Dentists working in the North East are significantly more likely to report being satisfied with the advanced NHS treatment of endodontics.

Question 11.5		How satisfied are you with the current advanced NHS treatment removable prosthodontics		Total
		Satisfied	Not satisfied	
Region of work	North East	31	18	49
	Rest of England	17	24	41
Total		48	42	90
Respondents removed from analysis as selected the option 'n/a - rarely refer for this treatment'				17

* satisfied is a composite score of responses 'very satisfied' and 'satisfied'
** not satisfied is a composite score of responses 'neither satisfied nor unsatisfied', 'unsatisfied' and 'very unsatisfied'

Pearson Chi Square: P= 0.015
Implication: Dentists working in the North East are significantly more likely to report being satisfied with the advanced NHS treatment of removable prosthodontics

**Question 11.7**		How satisfied are you with the current advanced NHS treatment for TMD		Total
		Satisfied	Not satisfied	
Region of work	North East	39	15	54
	Rest of England	21	21	42
Total		60	36	96
Respondents removed from analysis as selected the option 'n/a - rarely refer for this treatment'				10

* satisfied is a composite score of responses 'very satisfied' and 'satisfied'
** not satisfied is a composite score of responses 'neither satisfied nor unsatisfied', 'unsatisfied' and 'very unsatisfied'

Pearson Chi Square: P= 0.026
Implication: Dentists working in the North East are significantly more likely to report being satisfied with the advanced NHS treatment of TMD.

**Question 11.9**		How satisfied are you with the current advanced NHS treatment of anxiety/ psychosocial issues		Total
		Satisfied	Not satisfied	
Region of work	North East	31	18	49
	Rest of England	17	24	41
Total		48	42	90
Respondents removed from analysis as selected the option 'n/a - rarely refer for this treatment'				17

* satisfied is a composite score of responses 'very satisfied' and 'satisfied'
** not satisfied is a composite score of responses 'neither satisfied nor unsatisfied', 'unsatisfied' and 'very unsatisfied'

Pearson Chi Square: P= < 0.039
Implication: Dentists working in the North East are significantly more likely to report being satisfied with the advanced NHS treatment of anxiety/ psychosocial issues

**Question 12.1.**		How significant a barrier is ‘unclear pathway of referral’ to your current referral practises		Total
		Significant barrier*	Not a significant barrrier**	
Region of work	North East	36	22	58
	Rest of England	17	32	49
Total		53	54	107

* significant barrier is a composite score of responses ‘very significant barrier’ and ‘significant barrier’
** Not a significant barrier is a composite score of responses ‘no barrier’, ‘very insignificant barrier’ and ‘insignificant barrier’

Pearson Chi Square: P= 0.005
Implication: Dentists working in the North East are significantly less likely to think that ’unclear referral pathway’ is a significant barrier to current referral practises

**Question 14.1**		MCN’s would be beneficial for NHS patients		Total
		Agree*	Don’t agree**	
Region of work	North East	56	3	59
	Rest of England	40	9	49
Total		96	12	108

*‘Agree’ is a composite score of responses ‘strongly agree’ and ‘agree’
**’Don’t agree’ is a composite score of responses ‘neither agree nor disagree’, ‘disagree’, ‘strongly disagree’.’

Pearson Chi Square: P= 0.029
Implication: Dentists working in the North East are significantly more likely to agree that MCN’s would be beneficial for NHS patients.

Question 14.2		MCN's would be beneficial to GDPs		Total
		Agree*	Don't agree**	
Region of work	North East	56	3	59
	Rest of England	37	12	49
Total		93	15	108

*'Agree' is a composite score of responses 'strongly agree' and 'agree'
**'Don't agree' is a composite score of responses 'neither agree nor disagree', 'disagree', 'strongly disagree'.'

Pearson Chi Square: P= 0.004
Implication: Dentists working in the North East are significantly more likely to agree that MCN's would be beneficial for NHS patients.

Question 14.3		I would be interested in participating in a restorative Dentistry MCN		Total
		Agree*	Don't agree**	
Region of work	North East	46	13	59
	Rest of England	27	11	49
Total		73	35	108

*'Agree' is a composite score of responses 'strongly agree' and 'agree'
**'Don't agree' is a composite score of responses 'neither agree nor disagree', 'disagree', 'strongly disagree'.'

Pearson Chi Square: P= 0.011
Implication: Dentists working in the North East are significantly more likely to agree that MCN's would be beneficial for NHS patients.

Exploratory statistics: Number of years qualified

Below are all the instances where the independent variable 'years qualified' revealed a significant variation for the grouped responses to the questionnaire. For the purposes of this analysis area of work work split inmate two groups:

- 'Recent graduates' those who completed their primary dental degree between 2010-2019
- 'Older graduates' those dentists who completed their primary dental degree before 2010

**Question 11.1**		How satisfied are you with the current advanced NHS treatment for Implants		Total
		Satisfied	Not satisfied	
Year of graduation from primary dental degree	Recent Graduates	25	13	38
	Older Graduates	14	28	42
Total		39	41	80
Respondents removed from analysis as selected the option 'n/a - rarely refer for this treatment'				26

* satisfied is a composite score of responses 'very satisfied' and 'satisfied'
** not satisfied is a composite score of responses 'neither satisfied nor unsatisfied', 'unsatisfied' and 'very unsatisfied'

Pearson Chi Square: P= 0.004
Implication: Recently qualified dentists are significantly more likely to report being satisfied with the advanced NHS treatment of implants.

**Question 11.2**		How satisfied are you with the current advanced NHS treatment for endodontics		Total
		Satisfied	Not satisfied	
Year of graduation from primary dental degree	Recent Graduates	20	28	48
	Older Graduates	9	42	51
Total		29	70	99
Respondents removed from analysis as selected the option 'n/a - rarely refer for this treatment'				8

* satisfied is a composite score of responses 'very satisfied' and 'satisfied'
** not satisfied is a composite score of responses 'neither satisfied nor unsatisfied', 'unsatisfied' and 'very unsatisfied'

Pearson Chi Square: P= < 0.009
Implication: Recently qualified dentists are significantly more likely to report being satisfied with the advanced NHS treatment of endodontics.

**Question 11.4**		How satisfied are you with the current advanced NHS treatment for tooth surface loss		Total
		Satisfied	Not satisfied	
Year of graduation from primary dental degree	Recent Graduates	25	22	47
	Older Graduates	13	32	45
Total		38	54	92
Respondents removed from analysis as selected the option 'n/a - rarely refer for this treatment'				15

* satisfied is a composite score of responses 'very satisfied' and 'satisfied'
** not satisfied is a composite score of responses 'neither satisfied nor unsatisfied', 'unsatisfied' and 'very unsatisfied'

Pearson Chi Square: P= < 0.018
Implication: Recently qualified dentists are significantly more likely to report being satisfied with the advanced NHS treatment of tooth surface loss.

**Question 11.5**		How satisfied are you with the current advanced NHS treatment removable prosthodontics		Total
		Satisfied	Not satisfied	
Year of graduation from primary dental degree	Recent Graduates	31	18	49
	Older Graduates	17	24	41
Total		48	42	90
Respondents removed from analysis as selected the option 'n/a - rarely refer for this treatment'				17

* satisfied is a composite score of responses 'very satisfied' and 'satisfied'
** not satisfied is a composite score of responses 'neither satisfied nor unsatisfied', 'unsatisfied' and 'very unsatisfied'

Pearson Chi Square: P= 0.017
Implication: Recently qualified dentists are significantly more likely to report being satisfied with the advanced NHS treatment of removable prosthodontics

Question 15		Do you feel that you are already suitable to apply for a level 2 role?		Total
		Yes	No or Maybe	
Year of graduation from primary dental degree	Recent Graduates	10	42	52
	Older Graduates	26	30	56
Total		36	72	108

Pearson Chi Square value: P= 0.003
Implication: Recently qualified dentists are significantly less likely to feel already suitable for a level 2 role in a MCN.

Question 16.1		How important would a 'guaranteed patient flow' be for you when considering a role in an MCN		Total
		Important	Not important	
Year of graduation from primary dental degree	Recent Graduates	45	7	52
	Older Graduates	39	17	56
Total		84	24	108

* 'important' is a composite score of responses 'very important' and 'important'
** not important is a composite score of responses 'neither unimportant nor important', 'unimportant' and 'very unimportant'.

Pearson Chi Square value: P= 0.035
Implication: Recently qualified dentists are significantly more likely to feel that guaranteed patient flow is an important incentive for them when considering a role in a MCN.

Question 17.1		How much of a barrier would 'insufficient skill' be for you to take part in a MCN?		Total
		Significant barrier*	Not a significant barrrier**	
Year of graduation from primary dental degree	Recent Graduates	41	11	52
	Older Graduates	24	32	56
Total		65	43	108

significant barrier is a composite score of responses 'very significant barrier' and 'significant barrier'
** Not a significant barrier is a composite score of responses 'no barrier', 'very insignificant barrier' and 'insignificant barrier'

Pearson Chi Square value: P= <0.001
Implication: Recently qualified dentists are significantly more likely to think 'insufficient skill' is a significant barrier to them taking part in a MCN

**Question 17.2**		How much of a barrier would ‘access to appropriate training’ be for you to take part in a MCN?		Total
		Significant barrier*	Not a significant barrrier**	
Year of graduation from primary dental degree	Recent Graduates	40	12	52
	Older Graduates	30	24	56
Total		70	36	108

significant barrier is a composite score of responses ‘very significant barrier’ and ‘significant barrier’
** Not a significant barrier is a composite score of responses ‘no barrier’, ‘very insignificant barrier’ and ‘insignificant barrier’

Pearson Chi Square value: P= 0.020
Implication: Recently qualified dentists are significantly more likely to think ‘access to appropriate training’ is a significant barrier to them taking part in a MCN

**Question 17.4**		How much of a barrier would being ‘already too busy’ be for you to take part in a MCN?		Total
		Significant barrier*	Not a significant barrrier**	
Year of graduation from primary dental degree	Recent Graduates	21	30	51
	Older Graduates	35	21	56
Total		56	51	107

significant barrier is a composite score of responses ‘very significant barrier’ and ‘significant barrier’
** Not a significant barrier is a composite score of responses ‘no barrier’, ‘very insignificant barrier’ and ‘insignificant barrier’

Pearson Chi Square value: P= 0.027
Implication: Recently qualified dentists are significantly less likely to think ‘access to appropriate training’ is a significant barrier to them taking part in a MCN
